# Improved Nylon 6,6 Nanofiber Membrane in A Tilted Panel Filtration System for Fouling Control in Microalgae Harvesting

**DOI:** 10.3390/polym12020252

**Published:** 2020-01-21

**Authors:** Normi Izati Mat Nawi, Nur Syakinah Abd Halim, Leong Chew Lee, Mohd Dzul Hakim Wirzal, Muhammad Roil Bilad, Nik Abdul Hadi Nordin, Zulfan Adi Putra

**Affiliations:** Chemical Engineering Department, Universiti Teknologi PETRONAS, Seri Iskandar, Perak 32610, Malaysia; nomiizati@gmail.com (N.I.M.N.); nursyakinah94@gmail.com (N.S.A.H.); leongchewlee@gmail.com (L.C.L.); mdzulhakim.wirzal@utp.edu.my (M.D.H.W.); nahadi.sapiaa@utp.edu.my (N.A.H.N.); zulfan.adiputra@utp.edu.my (Z.A.P.)

**Keywords:** microalgae harvesting, nylon 6,6, nanofiber membrane, membrane fouling, membrane filtration

## Abstract

The competitiveness of algae as biofuel feedstock leads to the growth of membrane filtration as one of promising technologies for algae harvesting. Nanofiber membrane (NFM) was found to be efficient for microalgae harvesting via membrane filtration, but it is highly limited by its weak mechanical strength. The main objective of this study is to enhance the applicability of nylon 6,6 NFM for microalgae filtration by optimizing the operational parameters and applying solvent vapor treatment to improve its mechanical strength. The relaxation period and filtration cycle could be optimized to improve the hydraulic performance. For a cycle of 5 min., relaxation period of ≤2 min shows the highest steady-state permeability of 365 ± 14.14 L m^−2^ h^−1^ bar^−1^, while for 10 min cycle, 3 min. of relaxation period was found optimum that yields permeability of 402 ± 34.47 L m^−2^ h^−1^ bar^−1^. The treated nylon 6,6 NFM was also used to study the effect of aeration rate. It is confirmed that the aeration rate enhances the steady-state performance for both intermittent and continuous mode of aeration. Remarkably, intermittent aeration shows 7% better permeability than the full aeration for all tested condition, which is beneficial for reducing the total energy consumption.

## 1. Introduction

The world demand towards energy is increasing day by day. In the current situation, fossil fuels are widely used as the source of energy, but they are unsustainable, non-renewable, and non-environmentally friendly, since they release a massive amount of carbon and they have been blamed for being the main contributor to global warming. Thus, it is necessary for the researchers to give more attention toward renewable energy sources with high sustainability and environmentally friendly. This leads to the discovery of various sources of biofuels, including animal waste [1,2,3], agricultural residues [4], food waste [5,6], and biodegradable portion of industrial waste [7,8]. Although each of these approaches is being studied and applied to produce fuels, they are inadequate for meeting the global demand for liquid fuels.

Microalgae receive tremendous attention from the researchers and green-tech practiced organizations as the main source of biofuel feedstock. It can grow rapidly, even in harsh conditions without significant care on waste nutrients [9,10] and are able to accumulate generous amount of lipids [11]. Ideally, there are three stages required to produce biodiesel from microalgae namely; (i) cultivation of microalgae, to provide sufficient nutrition and retention before it is ready for harvesting, (ii) oil extraction via dewatering process; and, (iii) chemical modification of extracted oil to meet the desired final product [12]. Microalgae harvesting includes several techniques that involve coagulation and flocculation [13], flotation [14], centrifugation [15], and filtration [16], or a combination of various techniques [17,18].

Membrane filtration is seen as a promising technique for microalgae harvesting, since it provides almost complete retention of biomass and offers a very economical competitiveness when compared to other any energy-consuming methods [16]. Thus, membrane filtration has been intensively studied and explored for the algae harvesting. For instance, Zhang et al. [19] reported a study on developing an efficient membrane filtration system for algae harvesting with anti-fouling strategies. They also managed to develop a model to predict the flux decline and final concentration that was based on the resistance-in-series analysis and a cake development calculation. Besides, Yang et al. [20] also used ultrafiltration to harvest *Scenedesmus acuminatus* while using different feed directions of AF (bottom feed-top feed cycle) and BF (bottom feed). It was found that the average flux of AF with backwashing increased by 27.9% when compared to BF (68 L m^−2^ h^−1^). The result also shows that fouling was the most severe on the top section of the membrane fiber using BF due to a relatively low shear rate at the outlet. Meanwhile, in the AF direction, the frequent switching of flow direction enhances the shear rate along the fiber, hence improving its hydraulic performance.

Despite its advantages, harvesting microalgae biomass using membrane filtration is quite challenging due to small size of microalgae and density being close to water. Thus, the electrospun nanofiber membrane (NFM) was used for this study to address the challenges. Electrospinning is one of the reliable methods for synthesizing nanofibers with fiber diameter range of nanometers to micrometers. For the membrane material, nylon 6,6 have been chosen as the polymer, since it possesses great hydrophilicity, high tensile strength, and good mechanical properties, which depress the fouling propensity of the NFM [21,22]. However, the main drawback of the electrospun NFM is that it is low in mechanical strength [23]. This leads to the idea of conducting post-treatment on the fabricated membrane to improve its mechanical strength.

There are several techniques of post treatments that have been reported, which includes coating, blending, heat-pressed treatment, annealing, and solvent vapor treatment. These post-treatments alter the nanofiber properties and membrane structure, thus affecting the performance. For instance, Park et al. [24] coated polyvinylidene fluoride (PVDF) nanofiber support with polyvinyl alcohol (PVA) for forward osmosis and Liao et al. [25] attempted to blend the modified silica in the PVDF NFM to form a superhydrophobic membrane for membrane distillation (MD). Heat-pressed treatment is a process where the membrane was heat-pressed under certain temperature, pressure, and time. This method has been reported by Yao et al. [26] to treat the electrospun PVDF-co-hexafluoropropylene membrane to enhance membrane mechanical strength and liquid entry pressure, which greatly affects the hydraulic performance of MD. Yao et al. [27] also extended their study by implementing annealing onto the heat-pressed membrane. Xiang and Frey [28] reported a remarkable improvement of mechanical properties of nylon 6,6 NFM after treated via solvent vapor treatment.

The purpose of this study is to improve the mechanical properties of nylon 6,6 NFM for microalgae harvesting by employing solvent-vapor treatment. The solvent-vapor is preferred, since it is less aggressive to fiber linking and shows a trivial effect on the membrane morphology and dimension [29]. By using pristine nylon 6,6 NFM, the effect of relaxation rate on microalgae harvesting performance was investigated. In this study, we also attempt to find the optimum conditions for the treated NFM by supplying aeration and tilting the membrane panel at 20° to mitigate fouling with a different aeration mode; intermittent and continuous aeration.

## 2. Materials and Methods

### 2.1. Fabrication of Nylon 6,6 NFM

The membrane dope solution was prepared by dissolving 14 wt % of nylon 6,6 pellets (Sigma Aldrich, St. Louis, MI, USA in a mixture that has an equal weight ratio of formic acid (>98%, Merck, Kenilworth, NJ, USA and glacial acetic acid (99.85%, VWR Chemicals, Radnor, PA, USA). The dope solution was stirred while using a magnetic stirrer at least for 24 h to ensure the formation of homogenous solutions. An electrospinning set-up (Fanavaran Nano Meghyas, Tehran, Iran was used to electrospin the nanofiber mat. The homogeneous nylon 6,6 solution was filled inside a 5 mL syringe before being equipped with 0.6 mm inner diameter of needle tip and connecting to a high voltage electrode (26 kV). Afterwards, the solution was injected at a constant rate of 0.4 mL/h with a needle tip to screen the collector gap of 15 cm.

### 2.2. Post Treatment Using Solvent Vapor Treatment

After membrane fabrication, the nylon 6,6 NFM mat was exposed to solvent vapor as the post treatment. A 30 mL beaker of 98–200% concentration of formic acid was placed in a vacuum chamber at room temperature. The membrane pieces were exposed to formic acid vapor for 12 h. The membranes were dried at room temperature for at least 12 h before proceeding to the characterization and filterability test.

### 2.3. Membrane Characterization

The fabricated nylon 6,6 nanofiber membrane was characterized to determine the structural properties of the membrane. The surface microstructures of the membrane active layers were observed while using scanning electron microscopy (FESEM, Zeiss Supra55 VP, Felbach, Switzerland). ImageJ was used to measure the mean pore size, while the dry-wet method was applied to determine the porosity. Atomic force microscopy (AFM, NanoNavi E-Sweep Anton Paar GmbH, Graz, Austria ) was used to determine the surface roughness, while the surface contact angle was measured by using goniometer (OCA 20, Data Physics, Filderstadt, Germany) to gauge the hydrophilicity of the membrane surface. Finally, the mechanical strength of the membranes was determined while using Universal Testing Machine (UTM, Shimadzu, Nakagyoku, Kyoto, Japan), with a cross-head speed of 10 mm/min. that was guided by ASTM standard D638. The membranes were cut with a dimension of 30 mm × 70 mm and mounted with aluminum plates at both ends for a better grip.

### 2.4. Membrane Panel Assembly

Each pristine and treated nylon 6,6 NFM mats were assembled into a filtration panel (effective area of 165 cm^2^) to be applicable for filtration. The NFM were cut and then fixed onto a Novatexx 2471 non-woven to provide mechanical support by gluing all of the edges while using cyanoacrylate and epoxy glue (Hardex clear epoxy compound) to form a composite NFM before being further glued onto a panel frame. A spacer was placed in between the panel frame and the composite NFM in order to allow for permeate to flow. The low pressure inside the panel was created by the vacuum pump to force permeate to pass through the NFM from outside to the inside of the panel and the permeate line.

### 2.5. Chlorella Vulgaris Feed and Analysis

The Centre for Biomass and Biofuel Research Universiti Teknologi PETRONAS, Perak, Malaysia, provided the *Chlorella vulgaris* broth. It was used as received without pre-treatment. The broth was cultivated on the extract of compost as nutrient source and collected once a batch-wise cultivation reached the stationary phase, corresponding to biomass concentration of 1.1 g/L. The same broth was used for the entire tests for about two weeks and they were kept under constant aeration and illumination to somewhat maintain its condition.

### 2.6. Filtration Set-up

**Figure 1:**
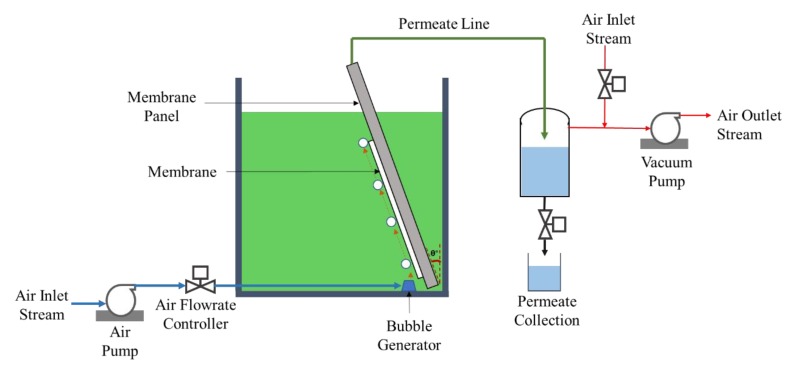
Illustration of the submerged filtration system.

The membrane filterability performance was evaluated while using a submerged and pressure-constant filtration system, as depicted in Figure 1 For the pristine NFM, the panel was placed in a filtration tank and positioned in the vertical condition, while the treated NFM was tilted to an angle of 20°. The trans-membrane pressure, △P was maintained at −0.1 bar for each experiment. The detail descriptions of the set-up and the filtration tests can be seen elsewhere [21]. The collected permeate was returned into the tank to maintain the feed liquid level.

The filtration flux and permeability of the membrane was determined while using Equations (1) and (2), respectively:

(1) $$J\;=\;\frac{V}{At}$$

(2) $$L\;=\;\frac{J}{\Delta P}$$

where *J* is water flux (L m^−2^·h^−1^), *V* volume of the permeate (L), *A* membrane effective area (m^2^), *t* effective filtration time (h), *L* permeability (L m^−2^·h^−1^·bar^−1^), and △*P* transmembrane pressure (bar).

## 3. Results and Discussion

### 3.1. Effect of Filtration Cycle on Hydraulic Performance of the Pristine NFM

**Figure 2:**
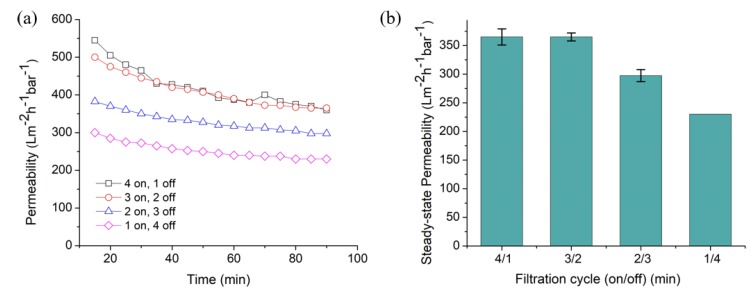
Effect of different filtration cycle on untreated nylon 6,6 NFM in five minutes total cycle (**a**) as function of filtration time with the steady state value summarized in (**b**).

Figure 2 shows the effect of relaxation cycle on permeability of pristine membrane for *C. vulgaris* filtration, which shows a remarkable effect. Relaxation is a type of physical cleaning by temporarily stopping the filtration to stop the drag force of the permeating fluid and release the accumulated foulant via back-transport. The application of relaxation cycle of ≤40% results in steady state permeability of 365 ± 14.14 L m^−2^·h^−1^·bar^−1^ and increasing the period of cycle reduces the permeability. Too long relaxation period reduces the filtration time and thus the permeate productivity in which permeate is produced. This finding is consistent with another report using a constant-flux system, in which a frequent short filtration period leads to high instantaneous flux [30].

The prolongation of relaxation period within a filtration cycle reduces the steady-state permeability of the membrane, as shown in Figure 2b The filtration/relaxation mode of 4/1 and 3/2 show a similar and highest steady-state permeability, which is about 365 Lm^−2^·h^−1^·bar^−1^. The results indicate that, in 5 min. filtration cycle period, relaxation of 1 to 2 min. is efficient for fouling control. When the relaxation time is further increased to 3 and 4 min., the steady-state permeability is respectively reduced by 18 and 37% due to less time provided for filtration activity.

**Figure 3:**
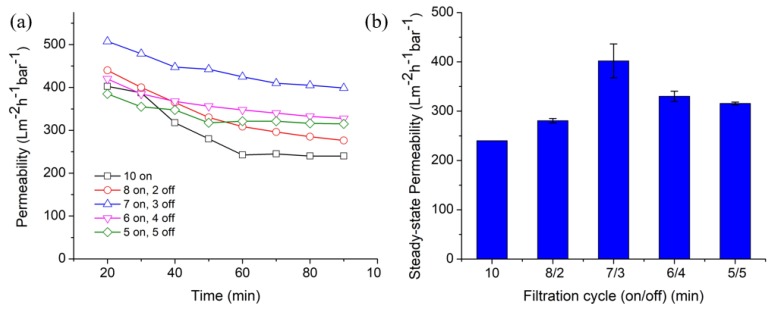
Permeability of untreated nylon 6,6 NFM under different filtration cycle involving relaxation in 10 min total cycle (**a**) as function of filtration time with the steady state value summarized in (**b**).

Figure 3a illustrates the permeability performance of the pristine NFM as function of time under 10 min. cycle of filtration/relaxation mode. The, “10 on” mode, representing the continuous filtration without relaxation, shows a great decline of permeability when compared to other modes. It proves that the continuous filtration mode faces highest tendency of fouling and gives the lowest steady-state permeability (Figure 3b). When relaxation period was introduced were up to 3 min, the permeability improves from 240 ± 0.25 up to 402 ± 34.47 L m^−2^·h^−1^·bar^−1^. This situation suggests that the optimum period of intermittent relaxation helps to reduce the compression of cake layer and maintain the high flux.

The steady-state permeability of the pristine NFM decreases to 330 ± 10.61 and 315 ± 2.65 L m^−2^ h^−1^ bar^−1^ when the relaxation time was prolonged to 4 and 5 min., respectively. In this case, the filtration/relaxation mode of 6/4 and 5/5 experience good fouling control, but their productivity is reduced due to short period of filtrations. From the results, we can conclude that 7 min. of filtration with 3 min. of relaxation is optimum and the most efficient mode for the 10 min. filtration cycle. A comparison between total cycle time of 5 and 10 min in Figure 2 and Figure 3 indicates that the latter offers higher overall permeability. This finding demonstrates that filtration and relaxation intervals need to be carefully adjusted to mitigate fouling without depressing the permeability.

The overall results show that optimizing filtration/relaxation cycle and the total cycle time can enhance the steady-state permeance of the pristine NFM. The steady state permeability value is higher than most reported the traditional phase invert ed membranes, as summarized in Table 1. The highest achieved permeability (402 ± 34.47 Lm^−2^·h^−1^·bar^−1^) is much higher than the pristine NFM reported earlier in which the steady state permeability was far below 300 L m^−2^·h^−1^·bar^−1^. These findings show that a simple operational parameter optimization yields remarkable improvement in hydraulic performance. However, a weak mechanical property remains the main drawback of the pristine NFM. Further exploration on parameters optimization could not be performed due to the damage on the NFM sheets. This finding has led us to explore the solvent vapor treatment technique to enhance mechanical property of NFM, as discussed in Section 3.2, and at the same time can maintain its hydraulic performance, as discussed in Section 3.3. The solvent vapor treatment is expected to allow the fibrous structure to be more compact and have higher mechanical strength. A smoother membrane surface is expected since this treatment promotes fusion and melting of overlapping fibers, which is advantageous for improving membrane fouling resistance and improving filterability performance.

### 3.2. Impact of Solvent Vapor Treatment of NFM Properties

**Figure 4:**
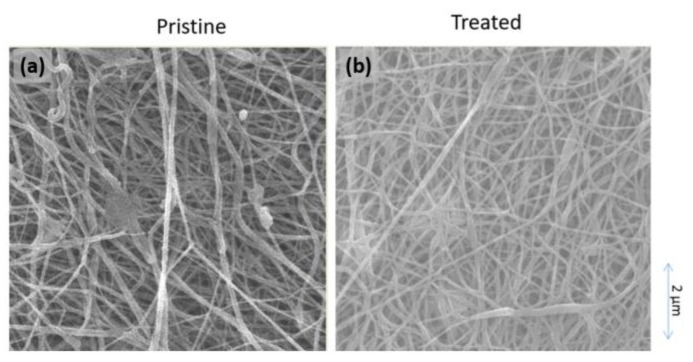
Field emission scanning electron microscopy (FESEM) image of the nanofiber membranes (NFMs) surface with magnification of 10,000× for (a) pristine and (b) solvent vapor treated nylon 6,6 NFM.

The surface morphologies of the pristine and treated nylon 6,6 NFMs show the swelling of the fibers for the treated one. The comparison of surface morphologies between the pristine and treated mats in Figure 4 show enlargements of the fibers for the treated mat (as indicated by the arrows in Figure 4b) suggesting the fiber swelling. The swelling phenomena occur due to the excessive solvent uptake and the dissolving of the polymer when exposed to the solvent vapor, which also leads to the fusion of the overlapping fibers [29,39].The crossing of overlapping fibers for the treated NFM leads to changes in other membrane properties, which might affect the filtration performance, as summarized in Table 2 The thickness, porosity, and mean pore size of the treated NFM are, respectively, lower by 18, 4, and 40% than the pristine NFM. The expansion of the fiber diameter due to the fusion and melting of fibers reduces the membrane pore size and depresses its porosity. On the other hand, it improves the mechanical strength more than two-fold (by 221%). It also reduces the surface roughness of the treated NFM by 63% from 231.10 ± 3.61 (pristine NFM) to 85.43 ± 2.30 nm and contact angle by 28%. This condition shows that the post-treatment of fiber mat by solvent vapor treatment that is able to improve hydrophilicity of the membrane, which is later expected to enhance the membrane fouling resistance.

### 3.3. Hydraulic Performance of the Solvent Vapor Treated NFM

The results in Section 3.2 show that the treated NFM possess better mechanical strength than pristine NFM, but at the same time poses weaker intrinsic properties (i.e., lower porosity, lower mean flow pore size). Thus, aeration was introduced to the system to delay fouling effect on top of parametric studies explored in Section 3.1 (filtration cycle and relaxation period). The air bubbles were expected to scour off the deposited particles or foulant by scouring it of the membrane surface, which is enhanced by the panel tilting configuration, as suggested by Eliseus et al. [34] to maximize the impact of air bubbles.

**Figure 5:**
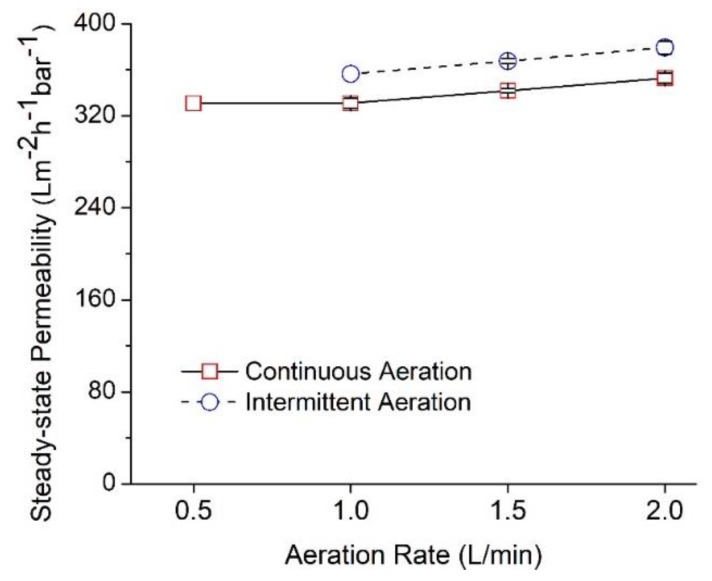
Effect of aeration rates on the steady-state permeability operated for 2 h. It was tested on continuous and intermittent mode of aeration with filtration/relaxation cycle of 3/2.

Figure 5 illustrates the steady-state permeability of the membrane filtration at different aeration rates with two modes of aeration: intermittent and continuous. It shows that increasing aeration rates enhance the steady-state permeability to reach the maximum value of 379.40 ± 5.60 L m^−^^2^ h^−^^2^ bar^−^^1^. This is in line with studies conducted by others [40,41], where a higher aeration rate produces larger number of bubbles, hence providing more intense contact with membrane surface, which helps to improve shear impact. Moreover, bigger bubbles that were produced at higher aeration rate also enhance fouling control. Despite having a positive impact on filtration performance, a higher aeration rate requires extra energy consumption, thus it is important to provide enough aeration without neglecting the aeration energy consumption.

In continuous aeration mode, the aeration rates of 0.5 and 1.0 L/min., two lowest rates applied in this study, show similar permeability, indicating that the air bubbles produced is too low and show minor effect for fouling control. We suspect that the intensity of air bubble released at rate 0.5 L/min. is too low to effectively remove the cake layer on the membrane surface.

Figure 5 shows that the intermittent aeration always poses a higher permeability of about 7% when compared to the continuous aeration mode. The intermittent aeration has been applied with a filtration mode of 1 min. on and 1 min. off for the filtration/relaxation cycle of 3/2. The finding seems to be contradictory to the notion that more bubbles impose better fouling control, because continuous aerations produce twice the number of bubbles of the intermittent one. Continuous air bubbling normally shows better performance in membrane fouling control than the intermittent bubbling, since the air bubbles continuously scour off the foulant [42]. Nevertheless, it is worth noting that the behaviour of air bubbles also varies, depending on the experimental setup, which might include feed type and properties, viscosity, range of aeration rate, type of membrane filtration, and position of membrane panel. To explain the finding in Figure 5, we propose a concept of optimum bubble number that leads to maximum permeability. Although the continuous aeration ensures the constant scouring of cake layer, the continual presence of the bubbles atop the NFM surface, unfortunately, might act as a cushion and hinder the contacts of feed liquid with the membrane surface. This will reduce the efficiency of the liquid flow toward the NFM surface. The results obtained in this study were found to be favourable to reduce energy consumption, since supplying aeration in intermittent mode enhances membrane permeability.

### 3.4. Stability of the Treated Nylon 6,6 NFM

**Figure 6:**
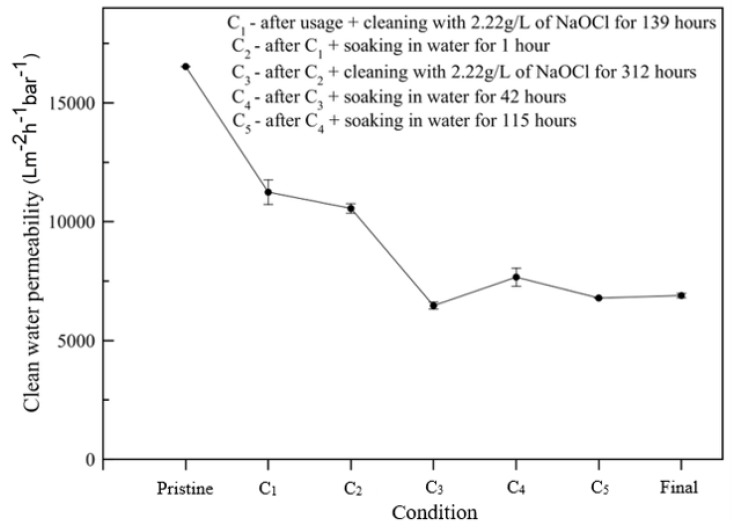
Performance of treated nylon 6,6 NFM during filtration of clean water after chemical cleaning using sodium hypochlorite solution.

Further study was performed to check the stability of the treated NFM, especially to gauge its resistance to the cleaning chemicals, after demonstrating good performance over 28 consecutive filtration tests for obtaining data in Figure 5 A series of treatments was conducted, and the results are depicted in Figure 6 Monitoring the clean water permeability (CWP) after series of filtrations and chemical treatments measured the stability.

The CWP of the pristine NFM shows the highest value, as it is the intrinsic property of the original membrane and it can be used as reference point of to what extent the membrane hydraulic performance decreases. As shown in Figure 6, after series of microalgae tests, the CWP significantly decreased from 16,500 to 12,500 L m^−2^·h^−2^·bar^−1^ at point C_1_ as the results of irrecoverable fouling from of the previous test, in which the foulant persists after such rigorous chemical cleaning of 2.22 g/L NaOCl for 139 h. Further soaking in water for 1 h at point C_2_ further decreases the CWP to 12,050 L m^−2^·h^−2^·bar^−1^.

Interestingly, a drastic decline of CWP is observed once further NaOCl solution cleaning was introduced at point C_3_ that lowers the CWP to 6800 L m^−2^·h^−2^·bar^−1^. The presence of NaOCl solution helps to remove the foulant deposited on the membrane surface, but at the same time, it might also disturb the structure of membrane, thus moderately affecting membrane filterability. Chemical cleaning while using NaOCl solution is one of the most common membrane cleaning agents. It used to eliminate most of the irreversible foulants on the membrane. However, the prolonged use of NaOCl has the potential to cause ageing on the membrane due to the oxidizing properties in the agent [43]. A study on the effect of oxidizing agent as membrane cleaning chemicals on the NFM shall be a focus in the future. Consecutive cleaning of the treated NFM while using water shows a constant final CWP of about 7000 Lm^−2^·h^−2^·bar^−1^, corresponding to 44% of the initial CWP value.

## 4. Conclusions

The optimization of relaxation period and filtration cycle was effective in enhancing pristine NFM hydraulic performance. The effect of the relaxation period for fouling mitigation under 5 min. filtration cycle shows that relaxation periods of ≤2 min. is the most effective to reduce compaction of cake-layer on the membrane surface. As for 10 min. filtration cycle, it was found that 7 min. of filtration with 3 min. of relaxation is the optimum cycle mode, which shows the highest steady-state permeability of 402 ± 34.47 L m^−2^·h^−1^·bar^−1^. However, weak mechanical strength hinders the prolonged use of a pristine nylon 6,6 NFM. The post-treatment of nylon 6,6 NFM was then conducted via solvent vapor exposure and it was proven to be capable of improving the membrane mechanical strength in expense of slight decrease in permeability. The filterability of the treated nylon 6,6, NFM was further assessed and it shows that the performance is better under higher aeration rate, despite the highest permeability of 378.40 ± 5.60 L m^−2^·h^−2^·bar^−1^ still being below the pristine one. Moreover, introducing aeration in intermittent manner further enhances the permeability performance (about 7%).

## Figures and Tables

**Table 1:** Hydraulic performance of membrane/system by implementation of fouling control system or newly developed membrane materials for microalgae harvesting.

Membrane Type/Fouling Control System	Membrane	Feed	Flux (Lm^−2^·h^−1^)	Permeance (Lm^−2^·h^−1^·bar^−1^)	Refs
Untreated NFM in tilted panel	Pristine nylon 6,6 nanofiber	1.1 g L^−1^ of *Chlorella vulgaris*	40.2	402.3	This study
Solvent vapor treated NFM in tilted panel	Treated nylon 6,6 nanofiber	1.1 g L^−1^ of *Chlorella vulgaris*	37.9	379.5	This study
Axial vibration and aeration	PVDF	0.3 g L^−1^ of *Chlorella pyrenoidosa*	238.4	340.6	[31]
Vibration and aeration	PVDF	0.08g L^−1^ of *Chlorella vulgaris*	32.5	325	[32]
Pristine NFM	Pristine nylon 6,6 nanofiber	1g L^−1^ of *Euglena sp.*	30.0	300.0	[21]
Backwashing and ventilation	PVDF	*Scenedesmus sp.*	130.0	260.0	[33]
Tilted panel	15% wt PVDF	1g L^−1^ of *Euglena sp.*	22.5	225.0	[34]
Membrane vibrations	9% and 12% wt PVDF	0.25 g L^−1^ of *Phaeodactylum tricornutum* 0.21 g L^−1^ of *Chlorella vulgaris*	± .21.25–42.5 ± 25.5–42.5	212.5–425.0 * 25–425.0 *	[16]
Axial vibration	PVDF	0.55 g L^−1^ of *Chlorella pyrenoidosa*	22.0–64.0 **	220–640.0	[35]
Disc type panel	PVDF	10 g L^−1^ of *Arthrospira (Spirulina) maxima*	57.0–142.9	95–238.3	[36]
Aeration in vertical panel	Cellulose ester	0.65 g L^−1^ of *Chlorella vulgaris*	11.6–20.5	23.2–41.0	[37]
Axial vibration membrane	PVDF	0.3 g L^−1^ of *Chlorella pyrenoidosa*	60.0	85.7	[38]

* The TMP is taken as 0.1 bar, ** Critical flux, NFM: nanofiber membrane, PVDF: polyvinylidene fluoride.

**Table 2:** Properties of the developed nylon 6,6 NFMs.

Parameters	Pristine NFM	Treated NFM
Thickness (mm)	0.22 ± 0.08	0.18 ± 0.02
Porosity (%)	71.30 ± 2.00	68.75 ± 0.45
Mean Pore Size (µm)	0.20 ± 0.03	0.12 ± 0.05
Average fiber diameters	138.5 ± 45.01	187 ± 141.3
Tensile strength (MPa)	737.56 ± 10.24	2373.27 ± 15.32
Surface roughness (nm)	231.10 ± 3.61	85.43 ± 2.30
Contact Angle (°)	56.01 ± 5.91	40.56 ± 5.29
Clean water permeability (Lm^−2^·h^−1^·bar^−1^)	18,701 ± 603	16,538 ± 254
